# Partial COVID-19 closure of a national park reveals negative influence of low-impact recreation on wildlife spatiotemporal ecology

**DOI:** 10.1038/s41598-023-27670-9

**Published:** 2023-01-13

**Authors:** Alissa K. Anderson, John S. Waller, Daniel H. Thornton

**Affiliations:** 1 School of the Environment, Washington State University, PO Box 642812, Pullman, WA 99164 USA; 2 National Park Service, Glacier National Park, PO Box 128, West Glacier, MT 59936 USA

**Keywords:** Conservation biology, Behavioural ecology

## Abstract

Human presence exerts complex effects on the ecology of species, which has implications for biodiversity persistence in protected areas experiencing increasing human recreation levels. However, the difficulty of separating the effect on species of human presence from other environmental or disturbance gradients remains a challenge. The cessation of human activity that occurred with COVID-19 restrictions provides a ‘natural experiment’ to better understand the influence of human presence on wildlife. Here, we use a COVID-19 closure within a heavily visited and highly protected national park (Glacier National Park, MT, USA) to examine how ‘low-impact’ recreational hiking affects the spatiotemporal ecology of a diverse mammal community. Based on data collected from camera traps when the park was closed and then subsequently open to recreation, we found consistent negative responses to human recreation across most of our assemblage of 24 species, with fewer detections, reduced site use, and decreased daytime activity. Our results suggest that the dual mandates of national parks and protected areas to conserve biodiversity and promote recreation have potential to be in conflict, even for presumably innocuous recreational activities. There is an urgent need to understand the fitness consequences of these spatiotemporal changes to inform management decisions in protected areas.

## Introduction

Fear of predators is a driving force of wildlife behavior and can lead to complex impacts that cascade across whole communities and ecosystems^1,2^. While much work has focused on the landscapes of fear created by apex predators, recent work also suggests that fear of humans can profoundly alter wildlife communities in a similar manner^3^. Although fear of humans may be an obvious expectation in areas with hunting, persecution, or other types of disturbance, fear effects can also manifest with more innocuous recreational activities such as hiking^4,5^. This is particularly concerning given that outdoor recreation is increasingly globally^6^, and even protected areas may be vulnerable to recreation-driven disturbance of wildlife communities^7^. Consequently, protected area managers now view human recreation as a major conservation issue^8^, albeit one with limited data to support management recommendations.

Despite the potential for simple human presence to alter mammal communities, responses documented to date are highly variable, with little ability to generalize regarding if and how human presence will exert an influence on species^9^. This high variability may be driven by several factors, including covariations between human presence and other types of disturbance (e.g., hunting or habitat destruction), varying levels of human presence, or species communities that consist of largely human-tolerant species. Moreover, most studies to date rely on observational data to determine how species are responding to human presence (e.g., analyzing the temporal or spatial patterns of species across a large landscape where there is a gradient of human activity, e.g.,^10–12^). While these types of studies contribute important knowledge, it can be difficult to disentangle if documented changes in wildlife are directly due to gradients in human presence or other unaccounted for factors that may covary with human activity. This is the same fundamental problem that affects co-occurrence modeling in general, where unmodeled factors may create a spatial association (or lack thereof) between pairs of species that is mis-interpreted as a positive or negative interaction^13,14^. Experimental manipulations of human activity are a potential way forward (e.g.,^3^), but difficult to conduct at landscape scales. However, the cessation of human activity that occurred in the early stages of the COVID-19 pandemic provides a ‘natural experiment’ in which to analyze the impacts of humans on wildlife^15,16^ while controlling for other environmental factors that may covary spatially with human presence. For example, by comparing differences in species spatiotemporal ecology when human activity is low during the closure and then increases afterward.

Glacier National Park (GNP) in northwest Montana, USA, experienced a drastic reduction of human activity during the summer of 2020 due to COVID-19 restrictions. The eastern half of the park was closed to the public, but typically receives a high level of summer recreation (primarily hiking). Unlike study areas for much of the existing literature on wildlife response to human recreation^10,17,18^, GNP is a relatively intact ecosystem harboring nearly a full suite of native mid- to large-sized mammals. Additionally, as a popular tourist destination GNP receives a very high level of visitation, often > 3 million people annually, mostly concentrated during summer months and on trails closest to roads. A COVID-19 closure within a popular, biodiverse and heavily protected National Park, provides an ideal situation to examine if and how presumably ‘low-impact’ recreation (i.e., hiking as the primary activity) affects wildlife.

Here, we examine how the spatiotemporal ecology of mid- to large-sized mammals is mediated by human recreation by examining data from camera traps located in eastern GNP, where cameras were placed in the exact same location and summer timeframe in a year that was closed to all recreation (2020), and a subsequent year with typical high levels of human recreation (2021). We predicted that apex carnivores would spatially avoid areas with high recreation (i.e., there would be a reduction in use of sites from 2020 to 2021, as the park transitioned from being closed to open to recreation) given the relative sensitivity of this group to human disturbance^3,19^. In response, we predicted that some mesocarnivore and ungulate species would exhibit increased detection and space use in areas with higher recreation, following mesocarnivore release (mesocarnivores can be ‘released’ from competition with apex predators and become more abundant;^20,21^) and human shielding theories (prey congregate in areas of high human activity to avoid risk from predation or competition from larger carnivores that are more human adverse;^22,23^). We also predicted that species would become increasingly nocturnal during the year with high recreation pressure (2021), echoing global trends in how species shift daily activity in response to human disturbance^24^.

## Methods

### Study area

**Figure 1:**
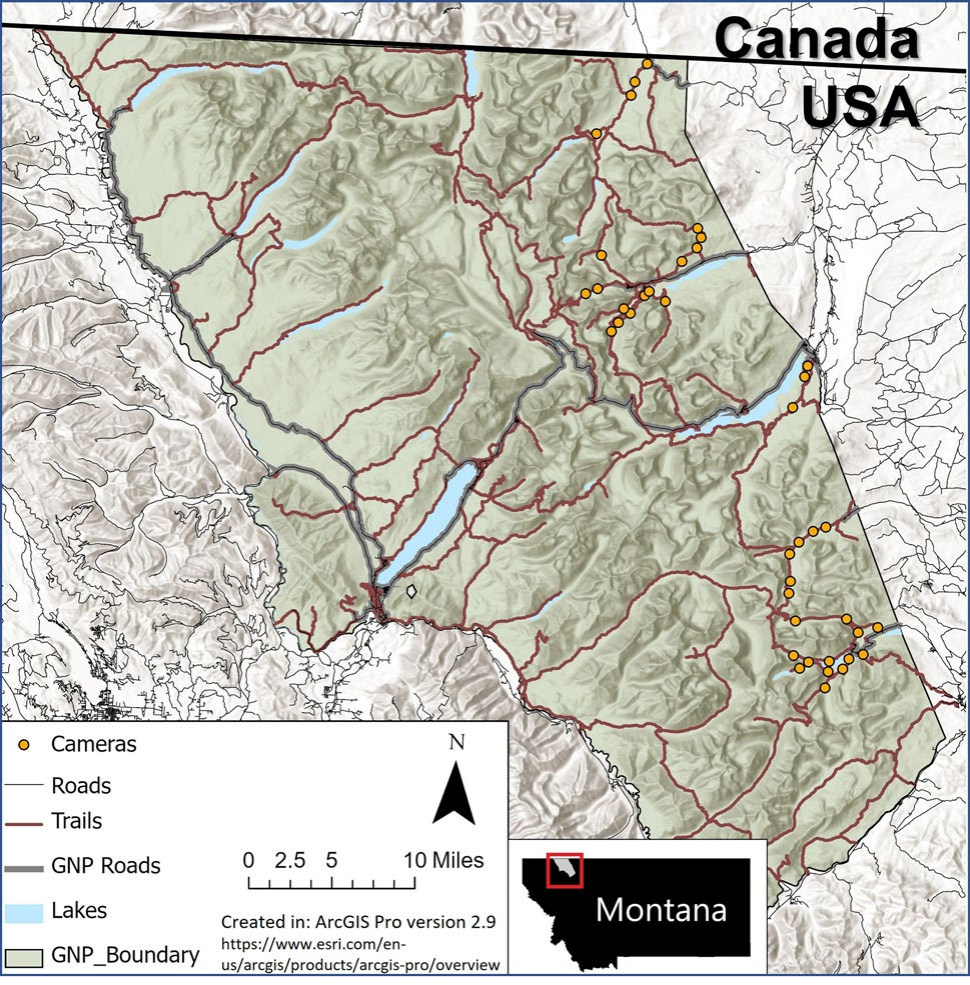
Camera locations on the eastern side of Glacier National Park, Montana. Cameras were located in the exact same location, and run during the same days, during a park closure with no recreation and when the park was open to recreation. Map made using ArcGIS Pro version 2.9 (https://www.esri.com/en-us/arcgis/products/arcgis-pro/overview).

GNP is a protected area covering 4100 ha in northwest Montana’s “Crown of the Continent” ecosystem (Fig. 1) and is an important part of the homeland of indigenous peoples including the Blackfeet, Kootenai, Pend d ’Oreille, and Salish tribes. In 1932, GNP joined Canada’s adjacent Waterton Lakes National Park to become the first international Peace Park, and the Waterton-Glacier International Peace Park was designated as a United Nations Educational, Scientific and Cultural Organization World Heritage Site in 1995. Bisected by the continental divide, the park forms headwaters draining into the Pacific Ocean, Hudson Bay, and the Gulf of Mexico. GNP borders provincial and national parks in British Columbia and Alberta, Canada, to the north, the Blackfeet Indian Reservation to the east, the Bob Marshall Wilderness Complex to the south, and the Whitefish Range managed mostly by the US Forest Service to the west.

The only activities allowed on trails in GNP are pedestrian or horseback travel. Bicycles and domestic dogs are prohibited on trails and resource extraction of any kind is prohibited park-wide. The purpose of closing the eastern half of GNP to visitors during the summer of 2020 was to prevent COVID-19 transmission to members of the Blackfeet Nation. Through mutual agreement, limited administrative access was allowed to National Park Service staff and permitted researchers (including this study) to drive across the reservation to access GNP, following strict travel regulations prohibiting stopping on the reservation to prevent COVID-19 transmission.

### Camera-trap sampling protocol

We used camera traps to document mammal and human spatio-temporal patterns. We set cameras in the same location during the 2020 eastside COVID-19 recreation closure and a normal year open to recreation (2021), with cameras spread out along a large portion of the eastern side of GNP (Fig. 1). Cameras were placed along hiking trails, located > 1 km from other cameras and attached cameras to trees roughly 0.5 m above trail height and 2–4 m off the trail to get a sufficient viewshed for detection. We set cameras to take a rapid-fire burst of 4 photos upon trigger followed by a trigger delay of one-minute. We only analyzed data from each camera site during periods when cameras were operational during both open and closed years. Thus, location, survey length, and survey dates (days and months) were replicated exactly. Environmental conditions were also comparable in the open vs closed years; there were no nearby wildfires and mean summer temperature and precipitation (measured at St. Mary, MT) differed only by > 1 °C and 10 cm respectively. Cameras were placed away from the few roads that provide access to the edges of the study area (all cameras except 2 were located > 500 m from roads), as well as from the few small backcountry camping sites (all cameras > 500 m away). Thus, the only major difference between the two years was the level of human recreation on hiking trails.

Once images were collected from cameras, we used MegaDetector machine learning software^25^ to separate photos containing animals, false triggers, and humans prior to expert sorting of images to species level. We used program R 4.0.2. (R Core Team 2021) and package ‘CamtrapR’ version 2.0.3^26^ to manage camera data. When creating detection histories for wildlife species for the dynamic occupancy models (see below), we used 10-day occasion intervals, whereby detection or non-detection of a species was calculated during each 10 days that a camera was active at a site.

### Influence of recreation on site use

To test for the influence of recreation on wildlife spatial distribution we ran dynamic occupancy models^27^, which required repeated estimates of detection/non-detection at a site. Dynamic occupancy models allow for an examination of extinction and colonization of sites (e.g., changes in occupancy at camera stations) between seasons, while accounting for imperfect detection at each site^27^. In this case, we were interested in changes in occupancy of sites between the first year that was closed to recreation and the second year that was open to recreation. These models estimate initial occupancy probability (ψ_t_; probability a site is occupied in the first season), colonization probability (γ_t_; the probability an unoccupied site in season t was occupied in season t + 1) extinction probability (ε_t_; the probability a site was occupied in season t but not in season t + 1), and detection probability (p_i_; probability of detecting the species given presence at the site during survey i). To estimate occupancy probabilities in subsequent seasons after the initial one, is simply the sum of a) the probability that a site was occupied and did not go extinct (ψ_t_ *(1 − ε_t_)) with b) the probability a site was unoccupied and was colonized ((1 − ψ_t_)* γ_t)_)). We note that occupancy models assume that the population is closed to changes in occupancy status during the sampling season^28^, which in our case, was the summer timeframe each year when cameras were running. This assumption was likely violated with highly mobile mammals. Thus, our dynamic occupancy modeling approach was assessing changes in ‘probability of use’ of camera sites, not changes in true occupancy^28^.

Given our relatively small sample size (40 sites in each year), and our focus on understanding changes in number of occupied sites between years, we fit simple dynamic occupancy models that did not include additional environmental covariates for initial occupancy, extinction, or colonization probabilities. Instead, we simply compared occupancy estimates between the two years of the study, one closed to recreation and one open to recreation, to examine how recreation impacted probability of site use. We did include a covariate for detection probability representing human recreation levels. We tested both a categorical variable representing closed vs open status (i.e., detection probability dropped across all camera sites equally when human recreation was allowed), and a continuous variable representing the number of human detections at a particular site (i.e., detection probability at a camera site was dependent on the level of human recreation at that specific site in the open year). For this later variable, we calculated the counts of human photos at each site to determine the recreation level. We considered time to independence as one-minute, meaning that if a human was photographed more than once at a camera within one minute only the first detection was included. We did not count individual people in photos; thus, our human detection data refers to numbers of times cameras were triggered by humans, whether by a group or by and individual. Hikers were the most common type of human detection (98%), followed by equestrians (2%), and we did not differentiate between these two types of use in our human detection data.

We compared a model with the categorical detection covariate, the continuous detection covariate, and a null model that had no covariates for detection using Akaike’s Information Criterion adjusted for small sample size (AICc). Occupancy models were fit in R package ‘unmarked’ version 1.0.1^29^. We assessed goodness of fit of the models using Mackenzie-Baily goodness of fit tests run with 1000 simulations using package ‘AICcmodavg’^30^.

Based on the best fitting model for each species, we calculated the conditional occupancy probabilities (occupancy estimates conditioned on the observed data) using the ‘ranef’ function in R package ‘unmarked’ for each camera in each year (open vs. closed to recreation). To estimate the expected number of sites occupied and 95% CIs in year 1 and 2, we used 10,000 bootstrap simulations (i.e., 10,000 random binomial draws to determine if each site was occupied or not based on the conditional occupancy estimate).

Because some species may still use sites in equal frequency (e.g., no change in number of sites occupied), but reduce the frequency of use when in the presence of human recreation, we used detection probabilities from the dynamic occupancy models as one metric to reflect differences in ‘intensity of use’ of camera sites. Given that our cameras were set in the exact same location and heights in the two years, any differences in detection were likely driven by changes in how many individuals were using the site and how active individuals were at the site rather than changes in camera placement methodology^12,31^.

As a second analysis of how human recreation impacted ‘intensity of use’ at each camera, we calculated the number of independent detections of each species at each camera trap during the course of the entire summer in a given year (note that this differs from the data used to estimate detection probability in the occupancy models, which just indicates detection or not in every 10-day interval). To calculate total number of independent detections, we considered time to independence as one-hour. To investigate how human recreation impacted seasonal detection counts, we then fit a negative binomial mixed model (with a log-link), with the count of the number of detections as the response variable and each camera trap as a random intercept to account for the repeated count in the open and closed year. For each species, we compared a model where number of detections was dependent on open/closed park status, one where number of detections was dependent on the rate of human detections at that site, and an intercept only model (number of detections constant between years, i.e., no effect of recreation). We selected a best-fitting model for each species with AIC. All analyses were conducted using the glmmTMB package in R^32^.

### Influence of recreation on temporal activity

To test if temporal wildlife activity patterns were influenced by recreation, we used nonparametric circular kernel density estimates to determine the temporal coefficient of overlap (∆) for each species between open and closed years using radian time in package ‘overlap’^33^. The temporal coefficient varies between 0 and 1, with 0 indicating complete separation of activity and 1 indicating complete overlap of activity^33^. We estimated precision of ∆ with 10,000 bootstrap simulations (i.e., resampling the open/closed dataset of detections for each species and calculating the subsequent overlap value;^33^) to estimate 95% confidence intervals. To determine if activity patterns differed significantly between samples we used the Watson’s Two-Sample test of homogeneity for circular data in package ‘circular’^34^ with a significance level of 5%^35,36^.

## Results

We had 40 cameras deployed on trails during the same date range in years open and closed to human recreation (Fig. 1). Including only dates when both open and closed cameras were operational, cameras were active for an average of 64.2 days (range: 33–85). During the open period average human triggers per day was 33.3 (range: 0.8–164.9) and during the closed period average human triggers per day reduced to 0.67 (range: 0.06–2.0; there was some detection of human activity due to limited administrative access to the area during the closure; Table 1). We detected 22 species of mid- to large-sized mammals in the two years, with sufficient detections of 14 species to fit dynamic occupancy models and calculate activity curves (Table 2). In total, we had 2503 and 2054 detections of those 22 species in the year closed and open to recreation, respectively, with 16 of 22 species having fewer detections in the year open to recreation. We detected wolverine (*Gulo gulo*), badger (*Taxidea taxus*), and mountain goats (*Oreamnos americanus)* in the year closed to recreation only, and raccoons (*Procyon lotor*) in the year open to recreation only (Table 2). We gathered > 100 detections for each of the 14 focal species except for wolves (*Canis lupus*; open n = 7, closed n = 15) and bighorn sheep (*Ovis canadensis*; open n = 58, closed n = 17), and moose (*Alces alces*) were the most detected species (open n = 212, closed n = 368) (Table 2).

**Table 1:** The mean number of daily independent detections (separated by more than 1 min) of human recreation at individual camera sites in a year closed to recreation (2020) and a year open to recreation (2021) in Glacier National Park.

Station	Daily human detections in closure year (2020)	Daily human detections in open year (2021)
1	0.06	7.24
2	0.64	49.92
3	0.33	58.92
4	1.14	12.82
5	2.02	23.77
6	1.87	9.25
7	2.00	18.57
8	1.05	73.67
9	0.17	113.51
10	0.21	59.06
11	0.62	41.73
12	0.37	56.05
13	0.24	34.09
14	0.71	71.38
15	0.88	164.89
16	0.79	11.86
17	0.78	9.69
18	0.72	9.37
19	0.09	0.98
20	0.08	0.89
21	0.89	31.00
22	0.42	84.01
23	0.38	6.39
24	0.49	5.70
25	0.25	6.71
26	0.79	25.06
27	0.64	12.47
28	1.16	21.42
29	1.03	7.00
30	0.20	5.78
31	0.11	1.07
32	0.33	4.91
33	0.31	4.62
34	0.45	54.39
35	1.57	81.63
36	1.44	76.38
37	0.24	11.93
38	0.33	6.96
39	0.59	8.89
40	0.59	47.54

Note that there was some human presence in 2020 due to administrative access.

**Table 2:** The number of independent detections (> 1 h separation of photos at a given camera) of mid- to large-sized mammals (> 1 kg in body mass) at remote cameras located in Glacier National Park in years closed and open to recreation.

Species	No. detections closed year	No. detections open year
American Badger (*Taxidea taxus*)	4	0
**Bighorn Sheep (Ovis canadensis)**	17	58
**Black Bear (*Ursus americanus*)**	199	112
**Cougar (*Puma concolor*)**	40	62
**Coyote (*Canis latrans*)**	294	78
**Elk (Cervus canadensis)**	130	62
**Grizzly Bear** (*Ursus arctos*)	156	137
Hoary marmot (*Marmota caligata*)	1	2
**Canada Lynx (*Lynx canadensis*)**	95	64
**Marten (*Martes americana*)**	82	32
**Moose (*Alces alces*)**	368	212
Mountain goat (*Oreamnos americanus*)	2	0
**Mule Deer (*Odocoileus hemionus*)**	284	271
Porcupine (*Erethizon dorsatum*)	4	1
Raccoon (*Procyon lotor*)	0	1
**Red Fox (*Vulpes vulpes*)**	33	266
**Snowshoe Hare (Lepus americanus)**	662	575
Striped Skunk (*Mephitis mephitis*)	9	1
**White-tailed Deer (*Odocoileus virginianus*)**	102	113
**Wolf (Canis lupus)**	15	7
Wolverine (*Gulo gulo*)	4	0
Yellow-bellied marmot (*Marmota flaviventris*)	2	1

Numbers are sums across all cameras. Bolded species had sufficient detections to be included in the occupancy and activity overlap analyses.

### Influence of recreation on site use

**Figure 2:**
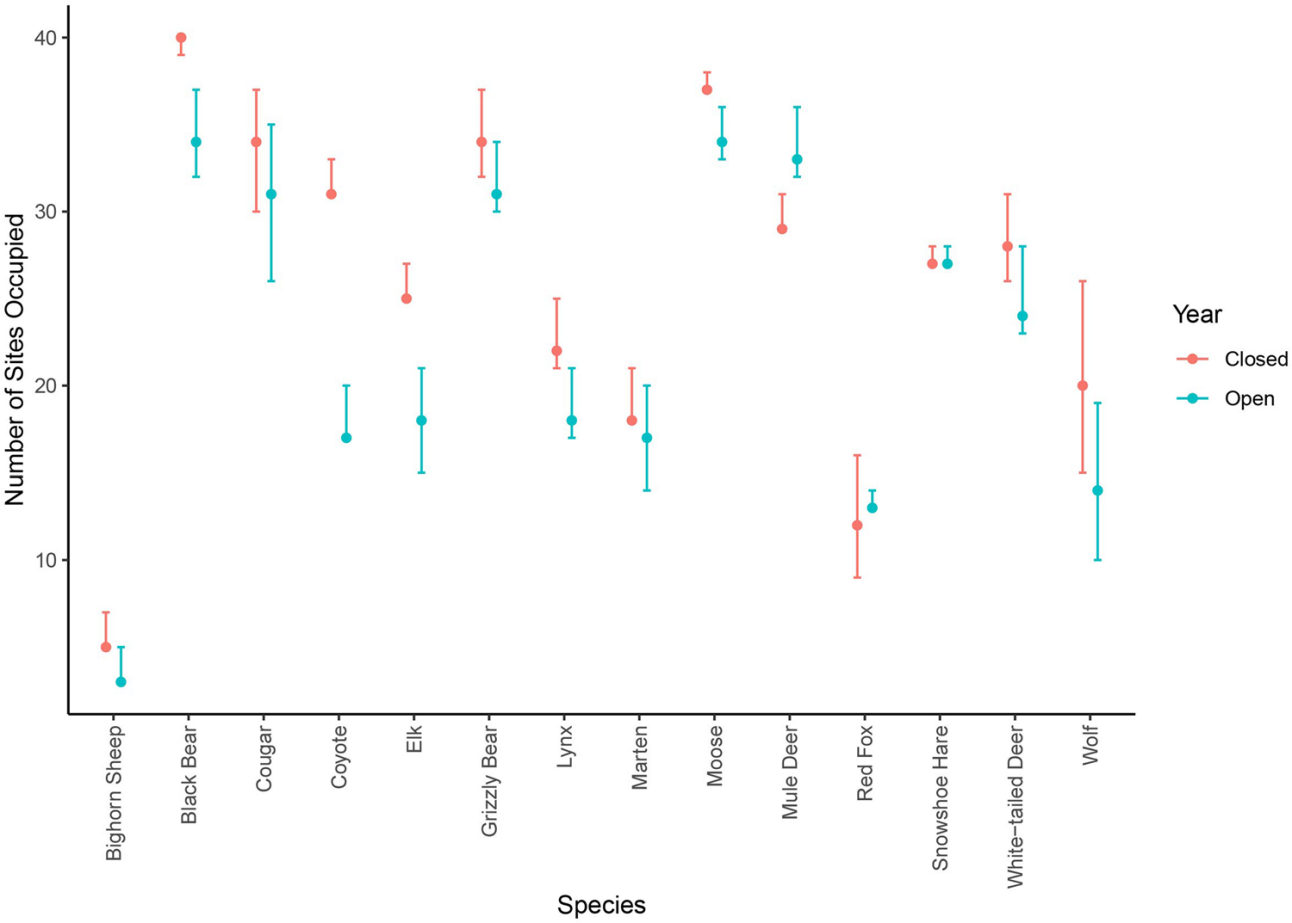
Estimated number of camera sites used (+ −95% CIs) by 14 mid- to large-sized mammals in a year closed to recreation and a year open to recreation based on dynamic occupancy models. Estimates from 10,000 bootstrapped simulations of presence/absence at sites based on conditional (latent) occupancy estimates of the best-fitting dynamic occupancy model for each species.

We found consistent negative effects of human presence on species, regardless of status as large carnivores, meso-carnivores, or herbivores. Based on best-fitting models, 12 of 14 species used a fewer number of sites in the year that the park was open to recreation (Fig. 2). Declines in use of sites were relatively modest but were 10% or greater for 6 of those 12 species (black bear, coyote, elk, lynx, white-tailed deer (*Odocoileus virginianus*), and wolves). Of the 2 species that occupied more sites in the open year (mule deer and red fox), neither increase was over 10% (Fig. 2). MacKenzie-Bailey goodness of fit tests indicated that models generally fit the data well, with c-hat values less than 1.5 for all species except wolves (2.56), the species with the lowest sample size.

The best-fitting models for 8 of 14 species included a covariate related to human presence for the detection process (either a categorical variable representing open vs. closed year or a continuous variable representing the number of human detections at each camera; Table 3). For 7 of those species, human presence exerted a negative impact on probability of detection (i.e., a negative impact on intensity of use of camera sites). Odds of detection declined in the year open to recreation by a factor of 0.60, 0.57, 0.58, and 0.67 for black bear (*Ursus americanus*), coyotes (*Canis latrans*), moose and mule deer (*Odocoileus hemionus*), respectively and by a factor of 0.82, 0.92, and 0.81 for every 10 additional triggers of a human at a camera for elk (*Cervus canadensis*), lynx (*Lynx canadensis*), and marten (*Martes americana*), respectively (Table 3). Only one species (red fox; *Vulpes vulpes*) displayed a positive association between detection probability and human presence, with detection increasing by a factor of 14 for every 10 additional triggers of a human at a camera.

**Table 3:** The influence of human recreation on intensity of use of sites (i.e., the degree to which a camera site was repeatedly used by a species), based on two surrogate measures: detection probability from dynamic occupancy models and the total number of independent detections based on negative binomial mixed models.

Species	Dynamic occupancy model	95% CI	Negative binomial model	95% CI
Bighorn sheep	–	–	0.15*human	(0.05, 0.25)
Black bear	− 0.11*human*	(− 0.19, − 0.03)	− 0.67*open	(− 0.96, − 0.39)
Cougar	–	–	–	–
Coyote	− 0.55*open	(− 1.06, − 0.05)	− 1.47*open	(− 1.90, − 1.03)
Elk	− 0.2*human	(− 0.36, − 0.04)	− 0.25*human	(− 0.43, − 0.07)
Grizzly bear	–	–	–	–
Lynx	− 0.08*human	(− 0.17, 0.01)	− 0.08*human	(− 0.16, 0.00)
Marten	− 0.22*human	(− 0.38, − 0.07)	− 0.71*open	(− 1.37, − 0.06)
Moose	− 0.55*open	(− 0.95, − 0.14)	− 0.59*open	(− 0.82, − 0.35)
Mule deer	− 0.39*open	(− 0.81, 0.02)	–	–
Red fox	2.66*human	(1.56, 3.77)	1.82*open	(0.81, 2.83)
Snowshoe hare	–	–	–	–
White-tailed deer	–	–	− 0.11*human	(− 0.23, 0.01)
Wolf	–	–	− 0.37*human	(− 0.84, 0.090)

Results show beta parameters from the best-fitting model for the influence of either a categorical variable representing when the park was open vs closed to recreation (denoted ‘*open’*; i.e., detection levels declined or increased equally across all cameras during the year open to recreation) or for a continuous variable representing the number of human detections at a camera (denoted ‘*human’*; i.e., detections declined or increased more at cameras with a greater number of human triggers in the open year). Species with no human covariates in the best-fitting model (i.e., no response of intensity of use to recreation) shown with dashed line.

*Beta parameter for dynamic occupancy models based on number of human triggers X 10.

When considering all detections of a species at a camera over the entire sampling interval (a second measure, in addition to detection probability above, of how intensely each camera site was used), 8 of 14 species had significantly fewer counts of detections in response to human recreation (i.e., negative parameter estimates from the negative binomial mixed model for the open year, or for higher levels of recreation at a camera; Table 3). This included large and mesocarnivores, as well as ungulates (black bear, coyote, elk, lynx, marten, moose, white-tailed deer, and wolves). Two species (bighorn sheep and red fox) had significantly greater counts of detections in response to recreation (Table 3).

### Influence of recreation on temporal activity

**Figure 3:**
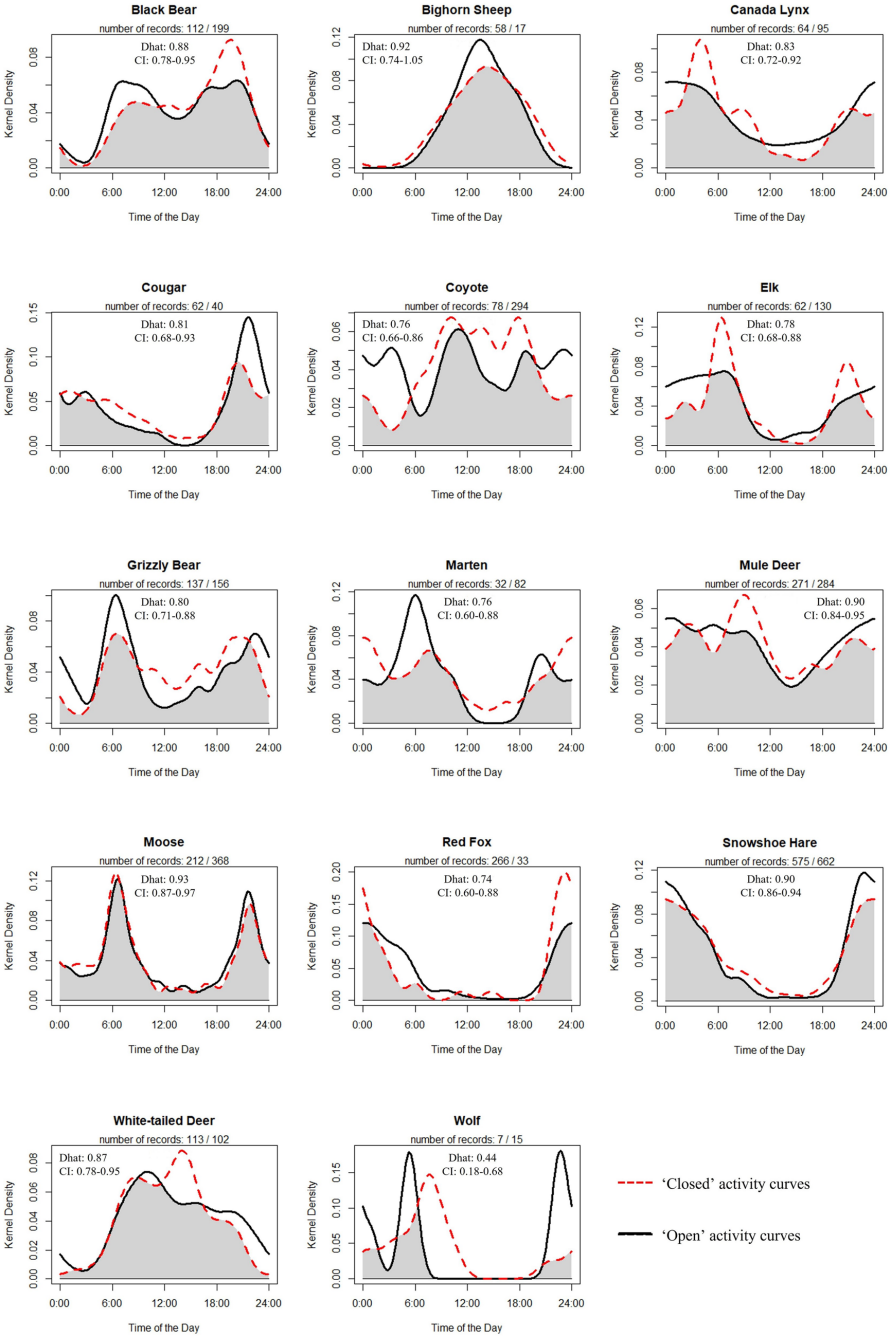
Daily activity overlap of each species between the year open and closed to human recreation. Dhat values represent the amount of overlap between the activity curves, with 0 representing complete separation and 1 representing complete overlap (95% CIs obtained via bootstrapping).

There was limited evidence of changes in daily activity patterns as a result of human presence. Effects were only seen with carnivores; grizzly bears, (*Ursus arctos*), coyotes, and red foxes had significant differences in temporal activity patterns between sites closed and open to recreation (Fig. 3). In agreement with our predictions, grizzly bears and coyotes had more diurnal activity during the closure and increased nighttime activity during the open period. For red fox, the closed period had more pronounced nocturnal activity peaks and also slightly more mid-day activity. Four additional species (cougar (*Puma concolor*), lynx, marten, and elk) had low overall coefficients of overlap (∆ < 0.85) between open and closed periods but were not found to show significant differences in activity due to small sample sizes that resulted in large confidence intervals. Of those species, cougar and marten displayed increased diurnal activity during the closure and increased nighttime activity during the open period, whereas elk displayed increased crepuscular activity during the closure (Fig. 3). Even several species with generally high coefficients of overlap (mule deer, white-tailed deer, snowshoe hare (*Lepus americanus*)) had less diurnal activity when the park was open to recreation based on a visual examination of the activity overlap graphs (Fig. 3).

## Discussion

The cessation of human activity that occurred with the COVID-19 pandemic has provided an unprecedented opportunity to examine how human activity impacts species mortality, behavior, and habitat use^37–40^. However, not all human activity is equal, with human presence in many places also associated with other forms of disturbance such as hunting or habitat modification^12^. Here, we found evidence that even low-impact human recreation (non-motorized, trail-based hiking) in a strictly protected national park affects the spatiotemporal ecology of a large variety of mid- to large-sized mammalian species. Although the influence of human presence on species was not strong in all cases, we found consistent negative responses across all groups of mammals, with fewer detections, reduced occupancy and detection probabilities, and generally decreased daytime activity. We document these effects even though there was likely ‘behavioral momentum’^41^ to overcome in how species used the GNP landscape, given that the decline of human activity with park closure was sudden and temporally constrained to one year. Although we cannot completely rule out the influence of human factors other than hiking in the patterns we documented (such as changes in traffic or camping that were drastically altered between years), the locations of our cameras away from other human infrastructure should have mitigated the influence of many of these. Moreover, because our design was a natural experiment where all other factors outside human hiking levels were controlled or varied little between years, our data do not suffer from confounding influences such as correlations between human recreation and other abiotic gradients that can plague observational studies of co-occurrence^13^.

The negative response of mammals to human recreation contrasts with some recent observational work finding highly mixed responses, (i.e., species responding both positively and negatively), to recreation or human presence^9,11,12^ but agrees with some other work taking advantage of the COVID-19 lockdown natural experiment^38,39^. Species responding negatively to recreation in our analysis include a mix of large and small carnivores and ungulates. Although with too few detections to model, several additional species of carnivore and herbivore, (e.g., wolverine, badger, and porcupines (*Erethizon dorsatum*), were detected more often during the closure when human presence was very low. The negative response across many types of mammals does not strongly support human shielding or mesocarnivore release theories as applied to human presence that formed the basis for one of our predictions, (with one notable exception discussed below). Relatively high levels of human use of the GNP landscape could be one factor leading to a more consistent negative response. We note that the areas of GNP where we conducted our study did not receive the highest levels of recreation in the park. Based on data collected from cameras located in other areas of the park, recreation levels can exceed 250 triggers per day, with our maximum at 160 triggers per day, suggesting even more pronounced effects could be seen elsewhere in the park.

Most species decreased diurnal activity in the presence of higher levels of recreation, generally supporting our predictions and previous research^17,24^, and this effect was most pronounced for carnivores. Given the strong proclivity for carnivore movement along roads and trails, alterations in timing of use of these structures may be a key mechanism of coexistence^12,42^. Across all species, there were fewer than expected significant differences in temporal activity, especially considering low coefficients of overlap between years with and without recreation for many species. However, the temporal analysis performed has been found sensitive to small sample sizes, thus we suggest caution when interpreting the significance values^43^.

We found mixed support for our prediction that apex predators would be negatively influenced by human recreation. Wolves appeared sensitive to recreation, whereas cougars were largely unaffected. Other studies have found negative response of recreation on cougar occupancy, especially when domestic dogs are present^18^ and a negative response to human development^17^. However, both of these disturbances were absent from our landscape. Combined with naturally nocturnal habits, cougars may have had limited need to alter spatiotemporal patterns in the presence of recreation. For the two other large predators, black bears and grizzly bears, we found contrasting effects of recreation. Black bear space use was influenced by human recreation but not temporal activity, and vice versa for grizzly bears. These patterns may be driven by the fact that where sympatric, black bears tend to be mostly diurnal, likely to avoid competition with grizzlies, which are mostly crepuscular/nocturnal^31,44^. Our finding that grizzly bears displayed increased diurnal activity in areas with lower levels of human recreation agrees with previous research^45^. For black bears, perhaps partly due to high temporal overlap with recreation, probability of detection and site use was negatively influenced. In GNP, where both species are abundant^46,47^, it is possible that subordinate black bears display temporal avoidance of dominant grizzly bears, and then spatial avoidance of high intensity recreation.

Although our results do not generally align with mesocarnivore release theories, red fox may be the exception as they responded positively to human recreation. Red fox response may be driven by recreation-induced changes in coyote ecology. Coyote occupancy, detection, and detection rate declined dramatically during the COVID-19 closure. This by itself is a surprising result, given coyotes general tolerance of human activity (e.g.,^7,43^, but again could be a sign of the level of recreation at our site or the experimental design allowing for more robust inference. Coyotes may spatially displace or kill red fox^48^, and large-scale analyses have found inverse relationships in their abundance^49^. Thus, the increase in red fox detection probabilities and detection rates with human recreation could be the result of a ‘release’ from competition with coyotes. Moreover, the increased use of daytime hours by fox when the park was open to recreation could also be a response to the increased nighttime activity of coyotes during the same period, a sign of behavioral release^43^. We note that overall use of sites by red fox was largely unchanged, so that it was intensity and timing of use that was changing. Outside of red fox and coyote, the other mesocarnivores we were able to model (martens and lynx) responded negatively in some aspect to recreation, with two other rare mesocarnivores (wolverines and badgers) only being detected at our cameras during the year of park closure.

Our results also did not align well with human shielding theories. Although mule deer were slightly more likely to use sites when the park was open, they had decreased probabilities of detection in the presence of recreation, suggesting mixed evidence for human shielding. Similarly, bighorn sheep were detected more times at cameras when the park was open but were also slightly less likely to use sites when there was recreation. Evidence for human shielding is further weakened by a strong negative response of the two largest bodied ungulate species to recreation. Elk decreased both site use and intensity of site use, consistent with some previous work^50^, with similar but less pronounced trends for moose. Even white-tailed deer had declining detection rates in the presence of human recreation. Taken together, our results suggest that at least for this one form of disturbance, and the scale at which we were measuring response (along trails), human shielding was not commonly occurring. Rather, these species were avoiding recreation similarly to carnivores. Another possibility is that exposure to human hunting or persecution outside the park for highly mobile individuals could be influencing their response to humans inside the park, though the degree to which individuals captured on our cameras are exhibiting transboundary movements is unknown.

As outdoor recreation is increasing in protected areas nationally and globally, assessing the impacts of presumably innocuous human presence on wildlife communities is essential to species management^51,52^. While outdoor recreation can benefit funding and support for conservation efforts^53^, increasing human presence from recreation can cause negative effects contrary to conservation objectives^8,54^. Most protected areas, National Park Service managed lands included, have a dual mandate to provide for conservation and recreation^55^. Our natural experiment quite clearly shows a reduction in site use and changes in the timing or intensity of activity in the presence of human hiking across a wide range of mammal species on our study area. Although other protected areas may not necessarily see the same effects due to context-specific factors such as overall levels of recreation, the fact that we observed this effect in an area where other kinds of human disturbance are virtually non-existent suggests a need to better understand the dynamics between recreation and wildlife to enable informed resource management decisions in protected areas. In addition, although we document substantial influence of recreation on the spatiotemporal ecology of a wide variety of mammals in GNP, we did not investigate how, or if, these responses impact fitness or population trends. Nor did we assess important aspects of this question such as seasonal changes in response to recreation, or the degree to which activity of species still occurs in the presence of recreation but shifts to nearby off-trail areas. We recommend that further studies focus on possible effects of recreation on wildlife fitness and the spatial extent of wildlife response to help managers better understand the tradeoffs associated with increasing recreation on public lands.

## Data Availability

The data used in these analyses are available upon request from authors Alissa Anderson or Daniel Thornton.
